# Supplementary material for: The Frequency of Fast Food Consumption in Relation to Wheeze and Asthma Among Adolescents in Gauteng and North West Provinces, South Africa
Source: Int J Environ Res Public Health. 2020 Mar 18;17(6):1994. doi: 10.3390/ijerph17061994 (PMC7143251; doi:10.3390/ijerph17061994)
Supplement: Supplementary file 1 [file ijerph-17-01994-s001.pdf]

Supplementary Table 1: Adjusted odds ratios of wheeze and fast foods among the study participants (N = 3641)

Characteristics	Odds Ratio	95% CI	P-Value
**Sex**			
Female	1	1	1
Male	0.76	0.53 – 1.36	0.376
**Type of house**			
Brick	1	1	1
Mud	1.69	0.95 – 2.89	0.074
Corrugated iron	1.31	1.01 – 1.70	0.048
Combination	0.80	0.51 – 1.28	0.363
Other	0.73	0.43 – 1.22	0.228
**Residential fuel type used for cooking/heating**			
Electricity	1		1
Gas	0.84	0.56 – 1.26	0.394
Paraffin	0.76	0.51 – 1.10	0.145
Open fires (wood or coal)	1.28	1.09 – 1.57	0.031
**Vigorous physical activity per week**			
Never	1	1	1
Once or twice per week	1.42	1.19 – 1.70	<0.001

Three times or more per week	1.60	1.32 – 1.97	<0.001
**Frequency of trucks passing near the residence on weekdays**			
Never	1	1	1
Seldom	1.20	0.93 – 1.56	0.158
Frequently	1.50	1.13 – 1.99	0.005
Always	1.43	1.14 – 1.79	0.002
**ETS exposure at home in the past 30 days**			
No	1	1	1
Yes	1.48	1.23 – 1.77	<0.001
**Do you smoke cigarette?**			
No	1	1	1
Yes	1.80	1.24 – 2.62	<0.001

1: Reference category

Supplementary Table 2: Adjusted odds ratios of asthma and fast foods among the study participants (N = 3641)

Characteristics	Odds Ratio	95% CI	P-Value
**Sex**			
Female	1	1	1
Male	0.71	0.40 – 1.21	0.497
**Type of house**			
Brick	1	1	1
Mud	3.13	1.74 – 5.62	<0.001
Corrugated iron	0.99	0.69 – 1.43	0.970
Combination	1.58	0.94 – 2.66	0.087
Other	0.46	0.18 – 1.17	0.105
**Residential fuel type used for cooking/heating**			
Electricity	1	1	1
Gas	1.26	0.78 – 2.02	0.348
Paraffin	0.66	0.38 – 1.14	0.133
Open fires (wood or coal)	1.44	1.28 – 1.69	<0.001
**Vigorous physical activity per week**			
Never	1	1	1

Once or twice per week	1.33	1.04 – 1.70	0.021
Three times or more per week	1.75	1.35 – 2.28	<0.001
**Frequency of trucks passing near the residence on weekdays**			
Never	1	1	1
Seldom	1.04	0.79 – 1.39	0.739
Frequently	0.95	0.66 – 1.36	0.767
Always	1.69	1.49 – 1.98	<0.001
**ETS exposure at home in the past 30 days**			
No	1	1	1
Yes	0.79	0.61 – 1.02	0.068
**Do you smoke cigarette?**			
No	1	1	1
Yes	1.74	1.12 – 2.71	0.015

1: Reference category
